# Ability Emotional Intelligence, Attachment Models, and Reflective Functioning

**DOI:** 10.3389/fpsyg.2022.864446

**Published:** 2022-05-02

**Authors:** Anna Maria Rosso

**Affiliations:** Department of Education, University of Genoa, Genoa, Italy

**Keywords:** ability EI, attachment models, reflective functioning, mentalization, emotion regulation

## Abstract

Previous studies have reported a significant positive association between ability emotional intelligence (EI) and attachment security. However, these studies may, to some extent, be misleading because they relied on self-report measures of attachment security. Furthermore, to our knowledge, no study has yet investigated the relationship between ability EI and mentalization, operazionalized as reflective functioning (RF), although EI and RF were assumed to be “conceptual cousins.” In an attempt to overcome some of the limitations of the previous research, the current study investigated the relationships between ability EI, attachment security, and mentalization measured *via* the Adult Attachment Interview (AAI). Ninety-three volunteer participants from an Italian community population (49.5% males), aged from 27 to 55 years (*M* = 39.44; *SD* = 6.84), took part in the study. Significant positive correlations were found between ability EI, attachment security, and RF. The results shed some light on the relationship between different attachment strategies and ability EI. Hyperactivating attachment strategies correlated negatively with ability EI, while the correlation between ability EI and attachment deactivating strategies depend on which defensive strategy is used: avoiding the painful emotional contact with the memory of unpleasant childhood attachment experiences positively correlated with experiential EI, whilst the resort to derogation of the attachment needs correlated with impairment in EI. Findings from the current study suggest that future studies in developmental psychology are needed to investigate the development of the ability EI in relation to the quality of the attachment models more in-depth.

## Introduction

Emotional intelligence (EI) is defined as a mental ability which makes it possible “to perceive emotions accurately, to use emotions to accurately facilitate thought, to understand emotions and emotional meanings, and to manage emotions in themselves and others” (Mayer et al., 2016, p. 291).

Allen and Fonagy (2006, p. 11) defined emotional intelligence and mentalization “conceptual cousins” in that both constructs pertain to identifying emotions in oneself as well as in other individuals, using emotions to organize thinking, understanding, and regulating emotions. In addition, both emotional intelligence and mentalization were found to be predictive of psychological health, the quality of the social relationship, and wellbeing (e.g., Lopes et al., 2003; Schutte et al., 2007; Martins et al., 2010; Zeidner et al., 2012; Karim and Shah, 2014; Ballespí et al., 2021). To our knowledge, despite the conceptual links between the two constructs, no empirical study has ever been carried out.

Within the multifaceted realm of mentalizing, Reflective Functioning (RF) is a specific construct related to the ability to be aware of the nature of mental states, to make an explicit effort to tease out mental states underlying behavior, to recognize the developmental aspects of mental states, and the mental states in relation to the interviewer in the context of autobiographical interviews which elicit the attachment system (Fonagy et al., 1998). From a developmental point of view, RF and secure attachment develop in close association (Fonagy et al., 2008), and moreover, mentalization was found to be significantly associated with attachment in childhood (Szpak and Białecka, 2020). A vast array of studies support the relationship between reflective functioning and attachment security (for a review, Luyten et al., 2019), while to date, the association between EI and attachment models has only been scarcely explored.

As Luyten et al. (2019) reported, the different attachment models are associated with distinct patterns of mentalization. Secure attachment has been associated with both high levels of mentalizing even in high arousal situations and with a rapid recovery of mentalization after its momentary loss, while preoccupied individuals, relying on attachment hyperactivating strategies, tend rather frequently in arousal contexts to lose mentalizing, after which they have a slow recovery (Luyten and Fonagy, 2015). Dismissing individuals, using attachment deactivating strategies, only apparently maintain controlled mentalizing longer and recuperate it rapidly after momentary failure (Vrtička et al., 2012), while actually, their performance in mentalizing measures depends on the level of the arousal brought out by the task. In this regard, it has been reported (Luyten et al., 2019) that the Adult Attachment Interview (AAI; Main et al., 2003) is a task which raises the level of arousal thereby putting a strain on the interviewee’s deactivating strategies by posing questions related to highly emotional autobiographical issues.

The few previous studies regarding the relationship between EI and attachment security (Kafetsios, 2004; Lanciano et al., 2012; Cherry et al., 2013; Altaras Dimitrijević et al., 2020) have reported that individual differences in ability EI are associated with quality of attachment, showing that securely attached individuals perform better on ability EI tasks, however, these studies might be flawed because they rely on self-report measures of attachment security. Kafetsios (2004) and Lanciano et al. (2012) used the four-item Relationship Questionnaire (RQ; Bartholomew and Horowitz, 1991), while Cherry et al. (2013) administered a short form of the Experiences in Close Relationships (ECR-SF; Wei et al., 2007), and Altaras Dimitrijević et al. (2020) used both the Modified Experiences in Close Relationship-Revised (M-ECR-R; Fraley et al., 2000) and the Revised Questionnaire for Attachment Assessment (QAA-R; Hanak, 2004, 2010). In addition, findings from these four studies are rather controversial; Kafetsios (2004) reported a positive correlation between ability EI and both secure and avoidant attachment, Lanciano et al. (2012) and Altaras Dimitrijević et al. (2020) found a significant and negative association between ability EI and both Anxiety and Avoidance attachment dimensions, whereas Cherry et al. (2013) found that attachment avoidance was significantly negatively correlated with total EI ability scores.

To overcome some of the limitations of the previous research, the current study investigated the relationships between Ability EI, attachment models, and mentalization measured *via* the AAI and the Reflective Functioning Scale (RFS). Based on the previously cited studies (Vrtička et al., 2012; Luyten and Fonagy, 2015), a negative association between EI and hyperactivating attachment strategies was expected, whereas the present study was exploratory regarding the association between ability EI and RF given that it is the first one investigating it. However, on the basis of the theoretical model outlined by Luyten et al. (2019), a positive significant relationship between ability EI and RF might be expected.

## Materials and Methods

### Participants

One hundred volunteer participants from an Italian community population agreed to participate in the current study, seven of whom were excluded from the analyses because of missing data or technical problems in recording the interview. The remaining group was made up of 47 females and 46 males ranging in age from 27 to 55 years (*M* = 38.58; *SD* = 7.67), with a level of education varying from 13 to 18 years (*M* = 15.60; *SD* = 2.50).

### Measures

The Mayer–Salovey–Caruso Emotional Intelligence Test (MSCEIT; Mayer et al., 2002) was administered to assess ability EI.

The MSCEIT is a 141-item ability measure of EI that consists of four branches: (a) emotion-recognition (this subscale includes 48 problems that require the subject to identify the emotion being expressed in photographs of faces or being evoked by photographs of landscapes); (b) emotion-facilitation (a subscale comprising 30 problems requiring the subject to identify the usefulness of a specific emotion in performing an activity, or the sensations associated with an emotion); (c) emotion-understanding (a subscale made up of 32 problems asking the individual to identify the cause of emotional reactions and to label complex emotions resulting from blended basic emotions); and (d) emotion-management (a subscale composed of 31 problems requiring the subject to choose effective ways to manage one’s own and others’ emotions in specific hypothetical situations).

The Mayer–Salovey–Caruso Emotional Intelligence Test provides five scores, one for each branch and one for total EI. Two additional scores can be used: an Experiential Emotional Intelligence (EEI) score which provides a measure of the ability to perceive emotions and to use them to facilitate thought, and a Strategic Emotional Intelligence (SEI) score which is an index of the ability to understand emotions and to use them purposefully for planning and self-management. SEI is regarded as a second order ability EI in that it implies more integrated and more cognitively complex abilities (Mayer et al., 2016).

Mayer et al. (2003) reported full-test split-half reliabilities of 0.91. The EEI and SEI reliabilities were 0.88 and 0.86, respectively. The reliabilities of the four branch scores (perceiving, using, understanding, and managing emotions) were between 0.76 and 0.91. In the current study, split-half reliability was 0.71 for full-test, 0.64 for EEI, 0.54 for SEI, 0.73 for Branch 1, 0.62 for Branch 2, 0.51 for Branch 3, and 0.44 for Branch 4.

The Adult Attachment Interview (AAI), rated in terms of both the Berkeley AAI System (Main et al., 2003) and the Reflective Functioning Scale (RFS; Fonagy et al., 1998), was administered.

The AAI is a semi-structured, hour-long interview designed to classify the state of mind with respect to early attachment experiences. The protocol consists of 18 questions. The interview begins by asking the subject to describe his/her relationship with their own parents in childhood. Then, the subject is requested to provide five adjectives that depict the relationship with each parent and for specific memories that would support the chosen adjectives. The next questions invite the subject to talk about their experiences of emotional distress, physical injury, illness, and separation from parents during their childhood. The subject is further requested to talk about possible experiences of rejection, abuse, maltreatment, and loss. The interviewees are also asked to reflect upon the impact of their childhood experiences on their personality and the mental states underlying their parents’ behavior. Finally, the interview shifts to the subject’s current relationship with his/her parents and the present relationship with his/her children, if any. The last question requires the individuals to say how experiences of being parented impact on their parenting.

The AAI includes nine nine-point scales for assessing relatively patterned or organized states of mind: coherence of transcript, idealization for the parent, insistence upon lack of recall, involved/involving anger, passivity of discourse, fear of loss, dismissing derogation, metacognitive monitoring, and overall coherence of mind.

Two additional scales assess unresolved/disorganized states of mind with respect to experiences of loss as well as experiences of abuse (including physical, sexual abuse, and extreme threats) by attachment figures. Disorganization and/or disorientation in thinking or discourse during discussion of a loss or an abuse are indexes of unresolved/disorganized states of mind.

A dimensional approach to the AAI was utilized, as suggested by recent studies (Bakermans-Kranenburg and van IJzendoorn, 2009; Whipple et al., 2011; Rosso and Airaldi, 2016) being the subscales “Idealizing toward mother,” “Idealizing toward father,” “Overall derogation of attachment,” “Derogation toward mother,” “Derogation toward father” markers of the dismissing state of mind, and the subscales “Passivity,” “Involving anger toward mother,” and “Involving anger toward father” indicative of the preoccupied state of the mind, while “Coherence of the mind” was considered the global index of security of attachment.

The RFS was designed to evaluate the capacity of mentalization in the AAI narrative since some questions in the AAI require reflective functioning (e.g., “Why do you think your parents behaved how they did during your childhood?”), while other questions permit reflective functioning (e.g., “Could you describe your first separation from your parents?”).

According to the scoring guidelines, “Awareness of the nature of mental states,” “Explicit effort to tease out mental states underlying behavior,” “Recognizing developmental aspects of mental states,” and “Mental states in relation to the interviewer” are the four markers of reflective functioning. After rating each identified passage of the AAI, an overall classification is assigned to the interview ranging from −1 (negative RF) to 9 (exceptional RF).

Validation studies of the RFS (Fonagy et al., 1998) showed discriminant and predictive validity, good inter-rater reliability, low correlations with education level, and no correlation with socioeconomic status and age. In this study no correlation emerged between participants’ RF and their education level (Spearman’s *rho* = 0.191, *p* = 0.143).

### Procedure

Examiners included four psychology graduate students trained by the author in administering the measures. Each examiner recruited 25 participants using a solicitation letter (available on request) written by the first author. The letter, which also served as an informed consent form, identified the project as one investigating the ability to recognize and manage emotions in autobiographic narrative as well in a non-autobiographic task.

In agreement with the statement in the letter, participants who signed the informed consent form were subsequently called by another examiner, who was not acquainted with them, to make an appointment.

Measures administration occurred at a time and place convenient for the participant. Examiners expressed gratitude for agreement to participate in the study, briefly described the project, assured the participant of confidentiality, and collected demographic data (age and education). The data were collected anonymously, each subject was assigned an identification number, and no compensation was provided.

The AAI and the MSCEIT were administered in a counterbalanced order during two sessions.

The Mayer–Salovey–Caruso Emotional Intelligence Test scoring was carried out online at the licensed publisher’s site, while the AAI protocols, which were initially audio-recorded and later transcribed verbatim, were rated in terms of both the Berkeley AAI System (Main et al., 2003) and the RFS (Fonagy et al., 1998) by the author as well as by an independent rater, both of whom were blinded to the MSCEIT scores. The inter-rater reliability was calculated: Pearson’s *r* for the AAI Scales for State of Mind ranged from 0.80 for the scale “Idealization of the relationship with the father” to 0.93 for the scale “Coherence of mind” (see Table 1). The inter-rater agreement for the overall classification of the RF scale was excellent with *k* = 0.86. All disagreements about overall classifications between the two raters were later discussed and clarified.

**TABLE 1:** Descriptive statistics, comparisons by gender, and inter-rater reliability.

	*N* = *93*		Females (*n* = 47)		Males (*n* = 46)				
	M	DS	M	SD	M	SD	*p*	*d*	*r*
Age	38.59	7.67	39.0	8.09	38.15	7.29	n.s.	n.s.	
Education	15.60	2.50	15.38	2.5	15.83	2.51	n.s.	n.s.	
EIT	87.42	9.30	90.29	9.18	84.57	8.62	0.003	0.64	
EEI	92.99	12.57	96.17	12.67	89.80	11.77	0.014	0.52	
SEI	86.32	5.93	88.17	5.64	84.43	5.67	0.002	0.66	
Branch 1	97.94	12.11	100.34	12.56	95.48	11.23	0.052	0.41	
Branch 2	90.24	13.33	93.15	13.42	87.26	12.69	0.032	0.45	
Branch 3	83.11	7.49	84.47	6.60	81.74	8.16	0.079	0.37	
Branch 4	91.06	7.45	93.04	6.70	89.04	7.61	0.009	0.56	
RF	3.34	1.31	4.00	1.10	2.67	1.16	<0.0001	1.18	
Coherence of mind	5.34	1.41	5.72	1.42	4.95	1.30	0.007	0.57	0.93
Passivity	2.27	1.74	2.26	1.74	2.28	1.76	n.s.	n.s.	0.88
Involving anger F	1.29	0.85	1.22	0.67	1.37	0.98	n.s.	n.s.	0.85
Involving anger M	1.68	1.27	1.87	1.45	1.48	1.03	n.s.	n.s.	0.89
Lack of recall	2.40	1.17	2.07	1.08	2.73	1.18	0.007	−0.58	0.91
Overall derogation	1.68	1.35	1.38	1.05	1.99	1.55	0.030	−0.47	0.89
Derogation F	1.39	1.12	1.15	0.67	1.63	1.40	0.040	−0.46	0.87
Derogation M	1.58	1.23	1.30	0.91	1.86	1.44	0.027	−0.48	0.91
Idealizing F	2.86	1.50	2.99	1.42	2.74	1.58	n.s.	n.s.	0.80
Idealizing M	3.39	1.65	3.16	1.57	3.63	1.72	n.s.	n.s.	0.89

*M, mean; SD, standard deviation; p, p-value; d, Cohen’s measure of effect size (| d| < 0.20: negligible; | 0.20| < d < | 0.50| : small; | 0.50| < d < | 0.80| moderate; d > | 0.80| : large); r, Pearson inter-rater reliability; EIT, MSCEIT total score; EEI, Experiential Emotional Intelligence score; SEI, Strategic Emotional Intelligence score; Branch 1, Perceiving Emotions; Branch 2, Facilitating Thought; Branch 3, Understanding Emotions; Branch 4, Managing Emotions; RF, Reflective Functioning Scale score; Passivity, Passivity of thought processes; Involving anger F, Involving anger toward father; Involving anger M, Involving anger toward mother; Lack of recall, Insistence on lack of recall; Derogation F, Derogation toward father; Derogation M, Derogation toward mother; Idealizing F, Idealization toward father; Idealizing M, Idealization toward mother.*

The approval of the ethics committee was not required because when the study was designed the local ethics committee had not yet been established. Even currently in the local institution the request for approval from the ethics committee is optional. All the procedures followed in the study were in accordance with the Helsinki Declaration of 1975, as revised in 2013, and in conformity with Italian law as established by the National Board of Italian Psychologists’ Code of Ethics.

## Results

Preliminary analyses of the data indicated that the study variables, except “Involving anger” and “Derogation,” were normally distributed with skewness and kurtosis values falling within the accepted range of ±2 (George and Mallery, 2010), thus appropriate for parametric statistical tests. Non-parametric statistical tests were used for the AAI Scales “Involving anger” and “Derogation.”

A general linear model was used to investigate the association of the variables of interest (MSCEIT, AAI, and RFS scores) with background variables (gender, age, and years of education). No significant correlation was observed between variables of interest, age, and level of education. Females obtained higher scores than males on MSCEIT, specifically on total scores, Experiential EI, Strategic EI, and Branch 4 “Managing emotion” with effect sizes in the moderate range. A large effect size was found regarding RFS scores with males reporting significantly lower scores than females. Descriptive statistics and comparisons by gender are reported in Table 1.

In the entire sample, Coherence of mind regarding attachment experiences correlated positively with MSCEIT total score, and with Experiential and Strategic EI, while all the AAI scales indicating the resort to hyperactivating attachment strategies (i.e., “Passivity of thought processes,” “Involving anger toward father,” and “Involving anger toward mother”) correlated negatively with MSCEIT total score. Scores on AAI scales, which are markers of the deactivating attachment strategies (i.e., “Insistence on lack of recall,” “Overall derogation,” “Derogation toward father,” “Derogation toward mother,” “Idealizing toward father,” and “Idealizing toward mother”), did not correlate with MSCEIT scores with the exception of “Derogation toward mother” which was found to be associated in the expected direction with MSCEIT total score, Strategic EI, and branch 4 “Managing emotion.”

Significant positive correlations were also found between RF and all ability EI scores but “Managing emotions” branch.

Since a significant association was found between gender and the variables of interest, correlation analyses were additionally performed separately for females and males. In the female subsample significant correlations were found in the expected direction between the AAI subscale “Passivity of thought processes,” the MSCEIT total score, the Experiential EI, and the Branch 1 “Perceiving Emotion.” The AAI subscale “Involving anger toward mother” correlated with the Strategic EI, and the Branch 1 “Perceiving Emotion.” The AAI Scale “Coherence of Mind” correlated with Strategic EI and Branch 3 “Emotion understanding.” A significant positive correlation was found between the AAI scale “Insistence on lack of recall” and Experiential EI, while RF correlated significantly and positively only with Branch 3 “Understanding emotions.”

Regarding the male subsample, the AAI scale “Involving anger toward father” correlated negatively with MSCEIT total score, Experiential EI, Branch 2 “Facilitating thought,” and Branch 3 “Emotion understanding.” The AAI scale “Involving anger toward mother” correlated negatively with MSCEIT total score, Strategic EI, Branch 3 “Emotion understanding,” and Branch 4 “Managing emotions.” Results are displayed in Table 2.

**TABLE 2:** Correlations between MSCEIT scores, AAI, and RFS scores.

	EIT	EEI	SEI	Branch 1	Branch 2	Branch 3	Branch 4
**Total sample (*N* = 93)**							
RF	0.296**	0.256*	0.311**	0.214*	0.219*	0.304**	0.135
Coherence of mind	0.277**	0.230*	0.289**	0.182	0.197	0.309**	0.107
Passivity of thought	−0.232*	−0.224*	−0.207*	−0.254*	–0.083	–0.088	–0.161
Involving anger F	−0.258*	−0.221*	−0.236*	–0.123	−0.234*	−0.208*	–0.135
Involving anger M	−0.237*	–0.185	−0.254*	−0.238*	0.020	–0.187	−0.212*
Lack of recall	0.035	0.120	–0.122	0.167	0.021	–0.139	–0.050
Overall derogation	–0.143	–0.072	–0.146	–0.014	–0.132	–0.144	–0.135
Derogation F	–0.130	–0.056	–0.160	0.012	–0.147	–0.202	–0.079
Derogation M	−0.216*	–0.132	−0.214*	–0.068	–0.180	–0.165	−0.225*
Idealizing F	0.017	–0.015	0.060	–0.090	0.087	0.084	0.001
Idealizing M	–0.070	–0.062	–0.066	–0.031	–0.113	–0.056	–0.044
**Females (*N* = 47)**							
RF	0.259	0.164	0.110	0.180	0.110	0.340*	0.200
Coherence of mind	0.221	0.171	0.298*	0.158	0.136	0.298*	0.053
Passivity of thought	−0.306*	−0.321*	–0.073	−0.345*	–0.130	–0.073	–0.216
Involving anger F	–0.077	–0.016	–0.172	–0.059	0.010	–0.034	–0.108
Involving anger M	–0.226	–0.183	−0.300*	−0.288*	0.110	–0.160	–0.247
Lack of recall	0.178	0.320*	–0.075	0.286	0.220	–0.113	–0.006
Overall derogation	0.038	0.049	0.047	0.006	0.078	–0.005	–0.031
Derogation F	0.147	0.132	0.149	0.067	0.107	–0.008	0.159
Derogation M	–0.072	–0.046	–0.052	–0.084	0.007	–0.060	–0.137
Idealizing F	–0.200	–0.239	–0.048	–0.190	–0.132	–0.094	–0.003
Idealizing M	–0.097	–0.043	–0.085	0.029	–0.170	–0.089	–0.110
**Males (*N* = 46)**							
RF	0.081	0.142	0.046	0.079	0.142	0.179	–0.175
Coherence of mind	0.203	0.176	0.196	0.105	0.155	0.257	0.018
Passivity of thought	0.170	–0.128	–0.197	–0.161	–0.033	–0.100	–0.120
Involving anger F	−0.371*	−0.357*	–0.252	–0.149	−0.427**	−0.312*	–0.096
Involving anger M	−0.389*	–0.248	−0.380**	–0.269	–0.206	−0.292*	−0.341*
Lack of recall	0.088	0.091	0.000	0.189	–0.040	–0.078	0.056
Overall derogation	–0.150	–0.047	–0.164	–0.037	–0.221	–0.183	–0.152
Derogation F	–0.190	–0.074	–0.232	0.026	–0.236	–0.263	–0.199
Derogation M	–0.201	–0.063	–0.236	0.017	–0.237	–0.202	–0.232
Idealizing F	0.172	0.153	0.109	–0.028	0.262	0.187	0.014
Idealizing M	0.031	–0.018	0.037	–0.036	0.001	0.014	0.078

*p < 0.05. **p < 0.01. EIT, MSCEIT total score; EEI, Experiential Emotional Intelligence score; SEI, Strategic Emotional Intelligence score; Branch 1, Perceiving Emotions; Branch 2, Facilitating Thought; Branch 3, Understanding Emotions; Branch 4, Managing Emotions; RF, Reflective Functioning Scale score; Passivity of thought, Passivity of thought processes; Involving anger F, Involving anger toward father; Involving anger M, Involving anger toward mother; Lack of recall, Insistence on lack of recall; Derogation F, Derogation toward father; Derogation M, Derogation toward mother; Idealizing F, Idealization toward father; Idealizing M, Idealization toward mother.

To explore the gender differences on RF and attachment model validity in predicting MSCEIT scores, a hierarchical multiple regression was performed, including gender as a dummy-coded variable (male = 0; female = 1) and its interaction terms with RF and attachment variables.

The MSCEIT score was the dependent variable (Total EI, EEI, and SEI), with gender at Step 1; RF, “Involving anger toward mother,” and “Insistence on lack of recall” at Step 2; and the three interaction terms gender × RF, gender × “Involving anger toward mother,” and gender × “Insistence on lack of recall” at Step 3. Scores of RF, “Involving anger toward mother,” and “Insistence on lack of recall” were mean centered before creating the product term.

Results are displayed in Table 3. The model explained 26.2, 22.3, and 26% of the variance of total EI, EEI, and SEI scores, respectively, while gender accounted for 9.5, 6.5, and 10%, respectively. “Involving anger” was the best predictor of both total EI and SEI, while “Insistence on lack of recall” was the best predictor of the SEI score. No moderating effect of gender on the relationship between RF, attachment models, and MSCEIT scores was found.

**TABLE 3:** Hierarchical multiple regressions.

	EIT						EEI						SEI					
	*Step 1*		*Step 2*		*Step 3*		*Step 1*		*Step 2*		*Step 3*		*Step 1*		*Step 2*		*Step 3*	
	*F* = 9.484		*F* = 6.896		*F* = 4.250		*F* = 6.245		*F* = 5.711		*F* = 3.437		*F* = 10–130		*F* = 6.662		*F* = 4.272	
	*p* < 0.01		*p* ≤ 0.0001		*p* ≤ 0.0001		*p* = 0.014		*P* < 0.0001		*p* = 0.003		*p* = 0.002		*p* ≤ 0.0001		*p* < 0.0001	
*Measures*	β	*p*	β	*p*	β	*p*	β	*p*	β	*p*	β	*p*	β	*p*	β	*p*	β	*p*
Gender	0.31	0.003	0.27	0.02	0.27	0.02	0.26	0.14	0.20	0.07	0.21	0.07	0.32	0.002	0.29	0.01	0.28	0.01
*Step 2*																		
RF			0.26	0.05	0.28	0.04			0.32	0.02	0.33	0.02			0.14	0.30	0.16	0.22
Anger			−27	0.01	−29	0.01			−16	0.11	−18	0.10			−0.33	0.00	−0.37	0.00
Lack			0.19	0.10	0.20	0.09			0.32	0.01	0.33	0.01			−0.04	0.72	−0.04	0.73
*Step 3*																		
Gender × RF					0.09	0.42					0.04	0.76					0.11	0.32
Gender × Ang					0.15	0.15					0.09	0.41					0.15	0.15
Gender × Lack					0.08	0.47					0.12	0.29					0.01	0.96
*R*2**	0.10		0.24		0.26		0.07		0.21		0.22		0.10		0.23		0.26	
Δ*R*2**			0.14		0.02				0.14		0.01				0.13		0.03	

## Discussion

The current study aimed to investigate the relationships between ability EI, attachment models, and Reflective Functioning since they are conceptually related psychological constructs (Allen and Fonagy, 2006). A significant negative association between attachment insecurity and ability EI was expected, especially with reference to the preoccupied attachment model, because preoccupied individuals resort to hyperactivating attachment strategies which lead to impairment in emotion regulation. The study was instead exploratory regarding the association between EI and RF, given that it was the first one in this field.

The further novelty of the current research is that it is the first study to apply the Adult Attachment Interview, which represents the gold standard for comprehensively assessing attachment strategies in adults.

Findings from the present study showed that RF correlated positively with all the MSCEIT scores supporting the hypothesis according to which they are distinct, albeit correlated, constructs.

No such correlations were observed in the male group after conducting the analyses separately for females and males. This result could be due to three possible, not mutually exclusive, explanations: (a) Correlation between RF and ability EI, albeit present, is weak; (b) The smaller sample size may have reduced the statistical power; (c) Males showed low RF scores and low MSCEIT scores, so in this group the variability was lower than in the female group.

In the female group, RF correlated with the ability to understand emotions (*r* = 0.340), that is the awareness of how emotions may change and combine. This finding could suggest that this ability implies an in-depth, authentic and embodied awareness of emotions, rather than mere intellectual knowledge. Thus, individuals who have greater access to their internal emotional life, being less defensive against them, might perform better on these tasks.

The gender differences both in RF and in ability EI, with males scoring lower than females, that was found in the current study replicated results recently reported by other scholars (e.g., Cabello et al., 2016; Jessee et al., 2016; Köber et al., 2019). Gender was a significant predictor of MSCEIT scores, however, no moderating effect on the relationship between RF, attachment models, and MSCEIT scores was found. Findings showed that “Involving anger toward mother” was the best predictor of both MSCEIT total and SEI score, while “Insistence on lack of recall” was the best predictor of EEI score, a finding which will be commented later.

After dividing the sample, it was found that in females the coherence of the mind regarding attachment experiences, which is the best index of attachment security, was positively and significantly associated with Strategic EI, and, particularly, with the ability to understand emotions. The same tendency was also observed in the males, but in this group correlations did not reach statistical significance. These expected findings corroborate the theoretical assumptions (for a review, Luyten et al., 2019) and replicated results from previous studies (Kafetsios, 2004; Altaras Dimitrijević et al., 2020). Again, the greater emotional openness that develops in (and is facilitated by) the context of the early secure attachment relationships, may provide, in adulthood, the possibility to access a greater range of emotions without the defensive need to avoid painful or overly exciting emotions.

Ability EI, both in males and in females, resulted more impaired in individuals who resort to preoccupied attachment strategies. During the AAI, they make the interviewer feel how intensely they are involved in their feelings of anger when talking about their childhood experiences and the relationship with their parents, so much so that it is difficult for them to maintain clear, fluent and coherent speech. Their sentences are often long and grammatically convoluted since their intense emotional involvement makes it difficult for them to express themselves clearly and concisely.

This expected finding was in line with some previous studies (Lanciano et al., 2012; Altaras Dimitrijević et al., 2020).

The unexpected result was that a dismissing strategy, namely, the insistence on lack of recall, correlated positively with experiential EI in the female group and, as stated above, was the best predictor of EEI in the entire sample. In the AAI, the interviewee insists on her inability to recall childhood episodes in an effort to block further queries. It is a defense aimed at avoiding the painful emotional contact with the memory of unpleasant experiences. This finding, although unexpected, was coherent with Luyten et al. (2019), who reported that dismissing individuals might lead clinicians to erroneously attribute a mentalizing ability to them because they are able to pseudomentalize (i.e., to perceive and use emotions at a mere cognitive level), while a more in-depth clinical observation makes it clear that, although their narrative can make extensive use of the mental state talk, it really lacks any affective grounding. In this regard, it is not surprising that “Insistence on lack of recall” scores correlated with Experiential EI but not with Strategic EI, being the former the lowest hierarchical level of ability EI (Mayer et al., 2002).

Findings from the current study could be useful to explain similar results obtained by Kafetsios (2004) and Lanciano et al. (2012). Conversely, Cherry et al. (2013) found a negative correlation between attachment avoidance and total EI scores. Unfortunately, a comparison between our findings and results obtained in previous studies is hardly possible as a vast array of studies have indicated an absence of relationship between self-reports of attachment styles and attachment organization as assessed by the AAI (for a review, Hesse, 2008; Crowell, 2014). Nevertheless, it could be argued that the correlation between ability EI and dismissing attachment strategies might depend on which dismissing strategies individuals prevalently resort to. Interestingly, our study showed that another dismissing strategy, namely, derogation toward attachment needs in relation to the childhood relationship with the mother, in the whole sample correlated negatively and significantly with EI total score, Strategic EI, and, specifically, with the highest level EI ability branch, i.e., “Managing emotions.” It could be argued that, while the insistence on lack of recall might be considered a more unemotional defensive strategy, derogation could imply a sort of “cold” anger that leads one to appear to be emotionally detached, but to actually be, on a more profound level, full of resentment which could not be expressed and felt at a conscious level, while still compromising the ability to manage the emotions.

On the basis of this supposition, the controversial findings that emerged from the previous studies about the correlations between dismissing attachment and ability EI could possibly be explained.

In conclusion, these results shed some light on the relationship between different attachment strategies and ability EI. Hyperactivating attachment strategies correlated negatively with ability EI, while the correlation between ability EI and attachment deactivating strategies depends on which defensive strategy is used: the avoidance of the painful emotional contact with the memory of unpleasant childhood attachment experiences positively correlated with experiential EI, whilst the resort to derogation of the attachment needs correlated with impairment in EI.

Findings from the current study suggest that future studies in developmental psychology are needed to investigate the development of the ability EI in relation to the quality of the attachment models more in-depth.

## Data Availability Statement

The raw data supporting the conclusions of this article will be made available by the authors, without undue reservation.

## Ethics Statement

Ethical review and approval was not required for the study on human participants in accordance with the local legislation and institutional requirements. The patients/participants provided their written informed consent to participate in this study.

## Author Contributions

AR designed the study, performed the analyses, wrote the manuscript, contributed to the article, and approved the submitted version.

## Conflict of Interest

The author declares that the research was conducted in the absence of any commercial or financial relationships that could be construed as a potential conflict of interest.

## Publisher’s Note

All claims expressed in this article are solely those of the authors and do not necessarily represent those of their affiliated organizations, or those of the publisher, the editors and the reviewers. Any product that may be evaluated in this article, or claim that may be made by its manufacturer, is not guaranteed or endorsed by the publisher.

## References

[B1] Allen J. G. Fonagy P. (eds) (2006). *The Handbook of Mentalization-Based Treatment.* New Jersey, NJ: John Wiley & Sons Inc 10.1002/9780470712986

[B2] Altaras Dimitrijević A. Jolić Marjanović Z. Dimitrijevićx A. (2020). A further step towards unpacking the variance in trait and ability emotional intelligence: the specific contribution of attachment quality. *Curr. Psychol.* 39 1340–1353. 10.1007/s12144-018-9837-3

[B3] Bakermans-Kranenburg M. van IJzendoorn M. (2009). The first 10,000 Adult Attachment Interviews: distributions of adult attachment representations in clinical and non-clinical groups. *Attach. Hum. Dev.* 11 223–263. 10.1080/14616730902814762 19455453

[B4] Ballespí S. Vives J. Sharp C. Chanes L. Barrantes-Vidal N. (2021). Self and Other Mentalizing Polarities and Dimensions of Mental Health: association With Types of Symptoms, Functioning and Well-Being. *Front. Psychol.* 12:566254. 10.3389/fpsyg.2021.566254 33613372 PMC7886987

[B5] Bartholomew K. Horowitz L. M. (1991). Attachment styles among young adults: a test of a four-category model. *J. Pers. Soc. Psychol.* 61 226–244. 10.1037/0022-3514.61.2.226 1920064

[B6] Cabello R. Sorrel M. A. Fernández-Pinto I. Extremera N. Fernández-Berrocal P. (2016). Age and gender differences in ability emotional intelligence in adults: a cross-sectional study. *Dev. Psychol.* 52 1486–1492. 10.1037/dev0000191 27570984

[B7] Cherry M. G. Fletcher I. O’Sullivan H. (2013). Exploring the relationships among attachment, emotional intelligence, and communication. *Med. Educ.* 47 317–325. 10.1111/medu.12115 23398018

[B8] Crowell J. A. (2014). “The Adult Attachment Interview,” in *The Routledge Handbook of Attachment: Assessment*, eds Farnfield S. Holmes P. (Routledge: Taylor & Francis Group).

[B9] Fonagy P. Gergely G. Target M. (2008). “Psychoanalytic constructs and attachment theory and research,” in *Handbook of Attachment: Theory, Research, and Clinical Applications., 2nd ed*, eds Cassidy J. Shaver P. R. (New York: The Guilford Press), 783–810.

[B10] Fonagy P. Steele H. Steele M. Target M. (1998). *Reflective Functioning Manual version 5.0 for application to Adult Attachment Interview.* England: University College London.

[B11] Fraley R. C. Waller N. G. Brennan K. A. (2000). An item response theory analysis of self-report measures of adult attachment. *J. Pers. Soc. Psychol.* 78 350–365. 10.1037/0022-3514.78.2.350 10707340

[B12] George D. Mallery M. (2010). *SPSS for Windows Step by Step: A Simple Guide and Reference, 17.0 update (10a ed.).* Boston: Pearson.

[B13] Hanak N. (2004). Construction of a new instrument for assessment of adult and adolescent attachment – QAA. *Psihologija* 37 123–142. 10.2298/PSI0401123H

[B14] Hanak N. (2010). Differences between pregnant women with secure and fearful attachment patterns with respect to transition to motherhood. *Psihološka istraživanja* 13 131–147. 10.5937/psistra1001131h

[B15] Hesse E. (2008). “The Adult Attachment Interview: protocol, method of analysis, and empirical studies,” in *Handbook of attachment: Theory, research, and clinical applications., 2nd ed*, eds Cassidy J. Shaver P. R. (New York: The Guilford Press), 552–598. 10.3389/fpsyt.2020.00243

[B16] Jessee A. Mangelsdorf S. C. Wong M. S. Schoppe-Sullivan S. J. Brown G. L. (2016). Structure of reflective functioning and adult attachment scales: overlap and distinctions. *Attach. Hum. Dev.* 18 176–187. 10.1080/14616734.2015.1132240 26754258

[B17] Kafetsios K. (2004). Attachment and emotional intelligence abilities across the life course. *Pers. Individ. Differ.* 37 129–145. 10.1016/j.paid.2003.08.006

[B18] Karim J. Shah S. H. (2014). Ability emotional intelligence predicts quality of life beyond personality, affectivity, and cognitive intelligence. *Appl. Res. Qual. Life* 9 733–747. 10.1007/s11482-013-9267-1

[B19] Köber C. Kuhn M. M. Peters I. Habermas T. (2019). Mentalizing oneself: detecting reflective functioning in life narratives. *Attach. Hum. Dev.* 21 313–331. 10.1080/14616734.2018.1473886 29768982

[B20] Lanciano T. Curci A. Kafetsios K. Elia L. Zammuner V. L. (2012). Attachment and dysfunctional rumination: the mediating role of Emotional Intelligence abilities. *Pers. Individ. Differ.* 53 753–758. 10.1016/j.paid.2012.05.027

[B21] Lopes P. N. Salovey P. Straus R. (2003). Emotional intelligence, personality, and the perceived quality of social relationships. *Pers. Individ. Differ.* 35 641–658. 10.1016/s0191-8869(02)00242-8

[B22] Luyten P. Fonagy P. (2015). The neurobiology of mentalizing. *Pers. Disord.* 6 366–379. 10.1037/per0000117 26436580

[B23] Luyten P. Malcorps S. Fonagy P. Ensink K. (2019). “Assessment of mentalizing,” in *Handbook of Mentalizing in Mental Health Practice*, eds Bateman A. Fonagy P. (Washington, DC: American Psychiatric Association Publishing), 37–62.

[B24] Main M. Goldwyn R. Hesse E. (2003). *Adult Attachment Scoring and Classification System, Unpublished Manuscript.* Berkeley: Department of Psychology University of California.

[B25] Martins A. Ramalho N. Morin E. (2010). A comprehensive metaanalysis of the relationship between emotional intelligence and health. *Pers. Individ. Differ.* 49 554–564. 10.1016/j.paid.2010.05.029

[B26] Mayer J. D. Caruso D. R. Salovey P. (2016). The ability model of emotional intelligence: principles and updates. *Emot. Rev.* 8 290–300. 10.1177/1754073916639667

[B27] Mayer J. D. Salovey P. Caruso D. R. (2002). *Mayer-Salovey-Caruso Emotional Intelligence Test: User’s manual.* Toronto: MHS.

[B28] Mayer J. D. Salovey P. Caruso D. R. Sitarenios G. (2003). Measuring emotional intelligence with the MSCEIT V20. *Emotion* 3 97–105. 10.1037/1528-3542.3.1.97 12899321

[B29] Rosso A. M. Airaldi C. (2016). Intergenerational transmission of reflective functioning. *Front. Psychol.* 7:1903. 10.3389/fpsyg.2016.01903 27999555 PMC5138206

[B30] Schutte N. S. Malouff J. M. Thorsteinsson E. B. Bhullar N. Rooke S. E. (2007). A meta-analytic investigation of the relationship between emotional intelligence and health. *Pers. Individ. Differ.* 42 921–933. 10.1016/j.paid.2006.09.003

[B31] Szpak M. Białecka P. M. (2020). Links between attachment and theory of mind in childhood: meta-analytic review. *Soc. Dev.* 29 653–673. 10.1111/sode.12432

[B32] Vrtička P. Bondolfi G. Sander D. Vuilleumier P. (2012). The neural substrates of social emotion perception and regulation are modulated by adult attachment style. *Soc. Neurosci.* 7 473–493. 10.1080/17470919.2011.647410 22217336

[B33] Wei M. Russell D. W. Mallinckrodt B. Vogel D. L. (2007). The Experiences in Close Relationship Scale (ECR)-short form: reliability, validity, and factor structure. *J. Pers. Assess.* 88 187–204. 10.1080/00223890701268041 17437384

[B34] Whipple N. Bernier A. Mageau G. A. (2011). A dimensional approach to maternal attachment state of mind: relations to maternal sensitivity and maternal autonomy support. *Dev. Psychol.* 47 396–403. 10.1037/a0021310 21171748

[B35] Zeidner M. Matthews G. Roberts R. D. (2012). The emotional intelligence, health, and well-being nexus: what have we learned and what have we missed? *Appl. Psycholol. Health Well Being* 4 1–30. 10.1111/j.1758-0854.2011.01062.x 26286968

